# In Vivo Comparative Evaluation of Biocompatibility and Biodegradation of Bovine and Porcine Collagen Membranes

**DOI:** 10.3390/membranes10120423

**Published:** 2020-12-15

**Authors:** Abdu Mansur Dacache Neto, Suelen Cristina Sartoretto, Isabelle Martins Duarte, Rodrigo Figueiredo de Brito Resende, Adriana Terezinha Neves Novellino Alves, Carlos Fernando de Almeida Barros Mourão, Jose Calasans-Maia, Pietro Montemezzi, Gilson Coutinho Tristão, Mônica Diuana Calasans-Maia

**Affiliations:** 1 Graduate Program, Dentistry School, Universidade Federal Fluminense, Niteroi 24020-140, RJ, Brazil; abdudacache@globo.com; 2 Oral Surgery Department, Dentistry School, Universidade Veiga de Almeida, Rio de Janeiro 20271-020, RJ, Brazil; susartoretto@hotmail.com; 3 Oral Surgery Department, Dentistry School, Universidade Iguaçu, Nova Iguaçu 26260-045, RJ, Brazil; resende.r@hotmail.com; 4 Clinical Research Laboratory in Dentistry, Universidade Federal Fluminense, Niteroi 24020-140, RJ, Brazil; 5 Post-Graduation Program in Dentistry, Universidade Veiga de Almeida, Rio de Janeiro 20271-020, RJ, Brazil; marcliodonto@terra.com.br; 6 Oral Surgery Department, Universidade Federal Fluminense, Niteroi 24020-140, RJ, Brazil; 7 Oral Diagnosis Department, Universidade Federal Fluminense, Niteroi 24020-140, RJ, Brazil; aterezinhanovellino@gmail.com; 8 Post-Graduation Program in Biotechnology, Universidade Federal Fluminense, Niteroi 24020-140, RJ, Brazil; mouraocf@gmail.com; 9 Orthodontics Department, Universidade Federal Fluminense, Niteroi 24020-140, RJ, Brazil; josecalasans@id.uff.br; 10 Private Practice, 24128 Bergamo, Italy; m.montemezzi@libero.it; 11 Periodontics Department, Universidade Federal Fluminense, Niteroi 24020-140, RJ, Brazil; gilsontb@vm.uff.br

**Keywords:** biocompatibility, biodegradation, collagen membrane, guided bone regeneration, subcutaneous, mice

## Abstract

Mechanical barriers prevent the invasion of the surrounding soft tissues within the bone defects. This concept is known as Guided Bone Regeneration (GBR). The knowledge about the local tissue reaction and the time of degradation of absorbable membranes favors the correct clinical indication. This study aimed to evaluate the biocompatibility and biodegradation of a bovine collagen membrane (Lyostypt^®^, São Gonçalo, Brazil) and compare it to a porcine collagen membrane (Bio-Gide^®^) implanted in the subcutaneous tissue of mice, following ISO 10993-6:2016. Thirty Balb-C mice were randomly divided into three experimental groups, LT (Lyostypt^®^), BG (Bio-Gide^®^), and Sham (without implantation), and subdivided according to the experimental periods (7, 21, and 63 days). The BG was considered non-irritant at seven days and slight and moderate irritant at 21 and 63 days, respectively. The LT presented a small irritant reaction at seven days, a mild reaction after 21, and a reduction in the inflammatory response at 63 days. The biodegradation of the LT occurred more rapidly compared to the BG after 63 days. This study concluded that both membranes were considered biocompatible since their tissue reactions were compatible with the physiological inflammatory process; however, the Bio-Gide^®^ was less degraded during the experimental periods, favoring the guided bone regeneration process.

## 1. Introduction

Several types of resorbable membranes are currently available on the market and have been tested in many studies in the field of periodontics and guided bone regeneration with great success. These membranes could be made from a group of natural polymers, such as collagen, and different types of synthetic polymers, such as aliphatic polyesters (e.g., polylactic or polyglycolic acid), or a combination of both [1,2].

Collagen comprises approximately 30% of the protein present in vertebrates and is present primarily in tissues with mechanical functions [3]. Type I collagen is the most abundant protein in connective tissue and can be found in the skin, tendons, bones, and teeth, which experience significant forces during daily activity [4]. Most manufactured membranes made with this type of collagen have different origins (bovine, porcine, or equine) or have differences in processing mode during manufacture, i.e., insertion of additional components and cross-linking methods, to obtain improvements in their properties, such as strength and biodegradability [5].

For implant and periodontal procedures in dentistry, an absorbable membrane of collagen is most widely used. It is a naturally occurring protein, has excellent biocompatibility, and has hemostatic and chemotactic characteristics for gingival fibroblasts and periodontal ligaments [6]. Further, it is resistant to tension, has controllable biodegradability, is noncytotoxic, has low antigenicity, and has excellent anti-inflammatory characteristics [4]. It is naturally reabsorbed by organisms, easy to manipulate, and has a molecular structure with little variance concerning the animal from which the collagen is extracted, leading to improved immunogenicity [7,8,9].

However, certain studies have reported that these membranes have unfavorable mechanical properties [10], such as rapid degradation under the action of collagenases, rendering it inadequate for use as a barrier [11,12]. Thus, such membranes do not maintain their mechanical integrity over long periods and often collapse at bone defects. Measuring this integrity is recognized as one of the most important requirements when selecting the type of resorbable membrane for use, especially in guided bone regeneration procedures [13,14].

Immediately after implanting a resorbable membrane, the body triggers a series of reactions to the injury, acting against the device placed. These include the blood–material interaction, the formation of a provisional matrix, acute inflammation, chronic inflammation, development of granulation tissue, foreign body reactions, and a fibrous capsule formation [15]. These events involve the attraction and activation of various cellular components, such as polymorphonuclear leukocytes, monocytes/macrophages, multinucleated giant cells, lymphocytes, or even resistant cells such as fibroblasts.

Thus, it is of fundamental importance to ascertain the membrane properties used for each case to facilitate planning and ensuring that the intended results can be achieved. Also, in this analysis, both membranes evaluated are commercially available; however, health professionals must have evidence that demonstrates the safety, efficacy, and biological activity of these products based on standardized protocols to reduce the use of inefficient outcomes. Thus, the presented study aimed to evaluate the biocompatibility and biodegradation of a bovine collagen membrane (Lyostypt^®^) and compare it to a porcine collagen membrane (Bio-Gide^®^) implanted in the subcutaneous tissue of mice, following ISO 10993-6:2016.

## 2. Materials and Methods

### 2.1. Experimental Groups

In this study, two membranes were evaluated: LT group, bovine collagen membrane (Lyostypt^®^, Laboratories B. Braun, São Golçalo, Brazil, test group) and BG group, porcine collagen membrane (Bio-Gide, Geistlich Pharma^®^, Wolhusen, Switzerland, test group). Also, the sham group (control group) was evaluated, in which the same procedures were performed, however, without membrane implantation.

Ethical Principles and Guideline Requirements

This study was approved by the Ethical Committee of the Universidade Federal Fluminense (CEUA/UFF), under protocol no. 869. The animal breeding and experiments were performed according to conventional guidelines of the NIH (National Institutes of Health) Guide for the Care and Use of Laboratory Animals following the Brazilian Directive for the Care and Use of Animals for Scientific and Didactic Purposes—DBCA and the CONCEA (Conselho Nacional de Controle e Experimentação animal) Euthanasia Practice Guidelines. Further, this study was carried out in compliance with the guidelines of the 3Rs program (reduction, refinement, and replacement), whose objective is to reduce the number of animals used during experimentation and to minimize their pain and discomfort (National Centre for the Replacement Refinement & Reduction of Animals in Research—NC3Rs, 2010). The experimental results were reported according to the ARRIVE (Animal Research: Reporting of In Vivo Experiments) [16] and Planning Research and PREPARE (Experimental Procedures on Animals: Recommendations for Excellence) [17] guidelines regarding relevant items.

### 2.2. Characterization of Animals

Thirty Balb-C mice, both male and female, at approximately 50 days old, weighing, on average, 20 to 30 g, were supplied by the Laboratory Animal Center (NAL) of the Universidade Federal Fluminense (NAL-UFF) for use in this study. The animals were randomly divided, through a random draw (using an opaque envelope containing the group name) by the principal investigator, into three groups: LT group, BG group, and the Sham group (control), without membrane implantation, (*n* = 10). The groups were subdivided according to experimental periods of evaluation (7, 21, and 63 days), with five animals in each group/experimental period.

The level of significance of 5% and a power test of 80% was used to calculate the sample size used in this study [Sealedenvelope. Available online: https://www.stat.ubc.ca/~rollin/stats/ssize/n2.html] (Adapted from Hassumi et al., 2018) [18]. The result suggested five animals in each group.

### 2.3. Welfare of Animals

Before, during, and after the experimental period, the animals were housed at the Laboratory of Animal Experimentation (LEA) of the Laboratory Animals Center (NAL) of the Universidade Federal Fluminense (UFF), in isolators created for this purpose.

Each isolator housed a maximum of five mice and was lined with dry wood shavings (pine shavings). They had a standard diet consisting of ground rations (Nuvilab^®^, Curitiba, Brazil), changed daily to mitigate the proliferation of fungi and bacteria. Water was administered at will through glass beakers with stainless steel spouts.

Special care and preparations were made to maintain the environment’s temperature to ensure the animals’ comfort, health, and welfare. This also facilitated the correct metabolic cycle for these experiments. The temperature of the environment was maintained between 20 and 22 °C, which is ideal for the animals’ growth and the control of the photoperiod, 12 h light and 12 h dark. A senior veterinarian conducted the nutritional parameters, animal care, and pre- and postoperative fasting.

### 2.4. Anesthesia and Surgical Procedures

After 24 h of fasting, all animals were put under general anesthesia intraperitoneally. The animals were immobilized with their bellies facing upwards. Their heads were tilted slightly downward to facilitate the viscera’s movement toward the diaphragm, reducing the risk of perforation of the intestines and cecum. A 21-G caliber needle (Becton-Dickinson^®^ Juiz de Fora, MG, Brazil) was used to inject 0.2 mL of the anesthetic solution in the animals’ lower left quadrant. The solution was prepared using 1.0 mL of 10% ketamine hydrochloride (Dopalen^®^-100 mg/mL Ceva, Paulina, SP, Brazil), 0.5 mL of 2% xylazine (Anasedan^®^-20 mg/mL Ceva, Paulina, SP, Brazil), and 8.5 mL of sterile saline solution (KabiPac^®^ Fresenius Kabi Brasil Ltd., Barueri, SP, Brazil).

Immediately after the pain reflex became absent, a trichotomy was performed with sterile razor blades and degermation using chlorhexidine degermant solution and 2% alcoholic chlorhexidine (Rioquímica, São José do Rio Preto, SP, Brasil) solution. Next, cloth from previously sterilized fields was used to delimit and isolate the surgical area [19].

Two rectilinear incisions in the skin were made on each side of the animal in the dorsal region, each measuring about 10 mm in length—one on the right side and one on the left side—using a no. 3 scalpel cable (Bard Parker^®^) blade no. 15C (Becton-Dickinson^®^ Juiz de Fora, MG, Brazil). Subsequently, divulsion was performed between the skin and the muscular fascia using blunt scissors (Metzenbaun 15-cm Colgran, São Caetano do Sul, SP, Brazil) until the subcutaneous tissue was exposed for posterior implantation of the membrane of equal sizes (1 cm).

**Figure 1:**
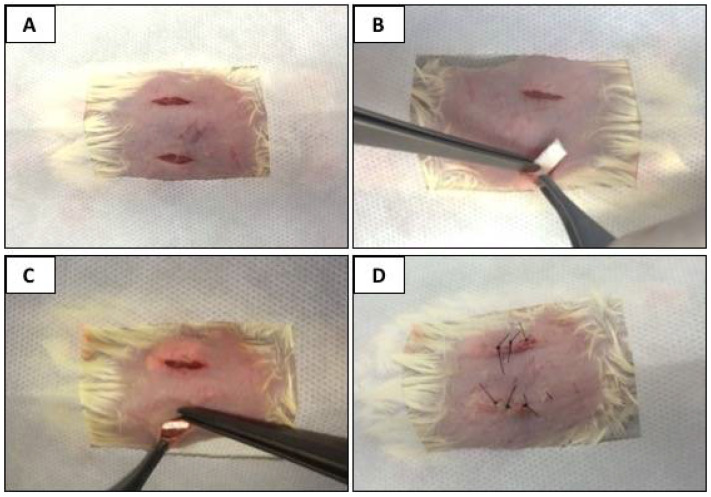
Surgical procedures. (**A**) Trichotomy, sterilized fields affixed, and two rectilinear incisions in the dorsal region of the animal. (**B**) After divulsion, the collagen membrane was implanted. (**C**) Membrane was accommodated in the subcutaneous tissue. (**D**) Suturing.

Five animals per group received two incisions (right and left side of the dorsum), and five animals received only one incision. In the right-side incision, the bovine collagen membrane (Lyostypt^®^ Laboratórios B.Braun, São Gonçalo, RJ, Brazil) was implanted. In the left-side incision, the porcine collagen membrane (Bio-Gide^®^ Geistlich, São Paulo, SP, Brazil) was implanted. The membranes were pre-immersed in a 0.9% sodium chloride physiological solution to facilitate manipulation and accommodation in the animals’ subcutaneous tissue. The incisions were sutured using nylon thread 5.0 (Ethicon^®^, Johnson and Johnson, São Paulo, SP, Brazil) and posterior antisepsis was conducted using gauze with an alcohol solution of 2% chlorhexidine (Rioquímica, São José do Rio Preto, SP, Brazil). This technique afforded protection to the surgical wound, reducing any possible secondary contamination. In the sham group, the same procedures (incision, displacement, and suturing) were performed; however, no collagen membrane was implanted (Figure 1).

According to their experimental group, the animals were maintained in isolators in the postoperative period (*n* = 5). The animals were strictly monitored until the anesthesia was wearing off to ensure that they were not injured due to poor motor coordination. After surgery, meloxicam 5 mg/kg (Medley, Curitiba, PR, Brazil) was administered subcutaneously every 24 h on the day of surgery and the subsequent two days. The animals were examined daily to evaluate and record any postoperative complications.

### 2.5. Obtaining the Samples

After the experimental periods of 7, 21, and 63 days, the five animals from each group received a lethal dose of 150 mg/kg pentobarbital and lidocaine for euthanasia. This was under the recommendations of National Resolution No. 13 of the National Council for the Control of Animal Experimentation (CONCEA). Samples were then taken from the sites of membrane implantation and also from the surrounding tissue. The Sham group also received a lethal anesthetic dose. Samples were then collected at the incision sites.

The samples were removed with a safety margin of approximately 5 mm on each side of the membrane implantation region using a no. 3 scalpel cable (Bard Parker^®^, São Caetano do Sul, SP, Brazil), blade no. 15C (Becton-Dickson^®^ Juiz de Fora, MG, Brazil), and the blunt end of a pair of scissors (Golgran^®^, São Caetano do Sul, SP, Brazil). All samples obtained were fixed in a 4% formaldehyde (phosphate buffer, pH 7.4) for a minimum of 24 h.

### 2.6. Material Processing

After being fixed, the samples were histologically processed via embedding in paraffin, cut into 5-µm-thick sections, and stained with Hematoxylin and Eosin (HE) for light microscopy assessment.

### 2.7. Microscopic Descriptive Analysis

A light-field microscope (OLYMPUS^®^ BX43, Tokyo, Japan) was used to facilitate a descriptive histological analysis of the slides. Selected images were captured through a microscope-coupled camera (OLYMPUS^®^ SC100, Tokyo, Japan) that used high-resolution software (CELLSENS^®^1.9 DigitalImage, Tokyo, Japan). A 40× increase was used for more comprehensive visualization of the area of interest and 200 and 400× magnification for obtaining higher cellular and tissue detail. In each slide, the presence, amount, and type of inflammatory infiltrate present at the membrane–tissue interface, neovascularization, fibrosis, connective tissue, and the implanted membrane’s degradation pattern were observed.

### 2.8. Evaluation of the Local Biological Effects of Implantation of the Biomaterials. Semiquantitative Histological Analysis: ISO 10993-6:2016/Part 6/Annex E

All microscopic analysis was performed by a single experienced pathologist who was blinded through coded slides. The digital images of the stained slides were obtained using a light-field microscope (OLYMPUS^®^, Tokyo, Japan). A semiquantitative histological analysis was performed on each subcutaneous slide from which 10 fields were scanned according to the area of interest, without any overlap, and captured using a high-resolution software (CELLSENS^®^1.9 DigitalImage, Olympus, Tokyo, Japan), with the objective of 400× by establishing a count of the surveyed elements, resulting in a score value indicating greater predominance among them. The biological response parameters at the tissue–membrane interface were evaluated and scored, as follows.

(1) The number and distribution of the inflammatory cell (neutrophils, lymphocytes, plasma cells, macrophages, and giant cells) as a function of distance from the material/tissue interface.
(2) The presence and the extension of necrosis.
(3) Inflammatory response parameters (neovascularization, the degree of fibrosis of the fibrous capsule, and fatty infiltrate).

This process was performed for each of the five animals investigated in all groups (LT, BG, and Sham) and the three experimental periods, 7, 21, and 63 days.

According to ISO 10993-6, when calculating each of the membrane materials’ total tissue response, the score values of inflammatory cell infiltrates (neutrophils, lymphocytes, plasma cells, macrophages, and giant cells). Necrosis was multiplied by 2 to expand the strength of value compared to the parameters of neovascularization, fibrosis, and fatty infiltration. The value was summed, and then an average for test groups and control was calculated.

The differences between values of the test (BG and LT) and control group (Sham) were ranked according to the following criteria: non-irritant (0.0 to 2.9), slight irritant (3.0 to 8.9), moderate irritant (9.0 to 15.0), and severe irritant (>15). The values were presented as median, minimum, and maximum.

### 2.9. Statistical Analysis

Statistical analysis to evaluate the difference between groups was performed. After the Shapiro–Wilk normality test, an analysis of variance (ANOVA) with Tukey’s post-test was applied. A *p*-value of <0.05 was considered significant. The Prism Graph Pad 8.3 software (La Jolla Inc., San Diego, CA, USA) was used. An independent statistician reviewed this methodology.

## 3. Results

### 3.1. In Vivo Response

The animals tolerated all the pre- and postoperative procedures very well, and no complications that could compromise the study results were detected.

### 3.2. Inflammatory Panorama Investigated after Subcutaneous Implantation

#### 3.2.1. Seven Days Post-Implantation

**Figure 2:**
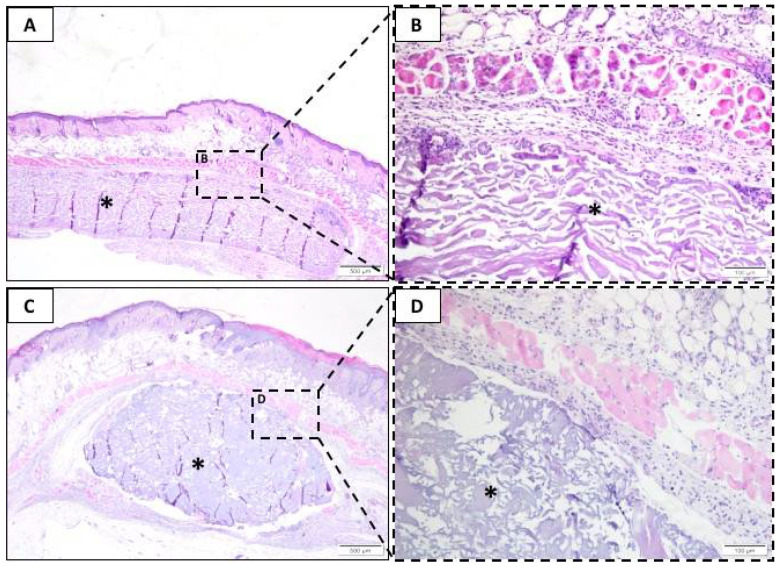
Representative photomicrographs of subcutaneous injury after seven days. Histological section stained with hematoxylin/eosin from the incision region in the BG groups (**A**,**B**) and LT groups (**C**,**D**). The area occupied by the membrane is indicated by an asterisk (*). (**A**,**C**) 40× magnification, scale bar: 500 µm; (**B**,**D**) 200× magnification, scale bar: 100 µm.

In the seven-day experimental period, the results of the cellular changes were observed in the groups tested, with the BG (Figure 2A,B) and LT (Figure 2C,D) results being similar. However, the LT presented a trend that increased in the presence of polymorphonuclear cells, lymphocytes, plasma cells, and macrophages surrounding the membrane. We also observed a large amount of the implanted membrane surrounded by connective tissue, focusing on moderate inflammatory infiltrate, predominantly mononuclear, in both groups (Figure 2). On average, there was little vascular proliferation, 1 to 2 capillary shoots per field in the BG and LT groups. In this period, the presence of multinucleated giant cells, fibrosis, fatty infiltrates, and tissue necrosis was not observed in any group.

The Sham group is not shown at all for experimental periods. The inflammatory values of Sham were used to present the semiquantitative results.

#### 3.2.2. Twenty-One Days Post-Implantation

**Figure 3:**
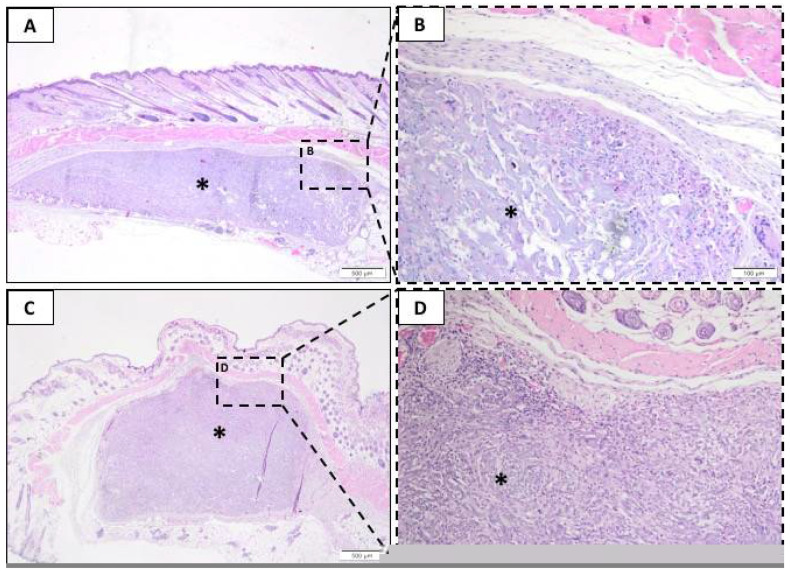
Representative photomicrographs of subcutaneous injury after 21 days. Histological section stained with hematoxylin/eosin from the incision region in the BG groups (**A**,**B**) and LT groups (**C**,**D**). The area occupied by the membrane is indicated by an asterisk (*). (**A**,**C**) 40× magnification, scale bar: 500 µm; (**B**,**D**) 200× magnification, scale bar: 100 µm.

After 21 days, the BG (Figure 3A,B) and LT (Figure 3C,D) groups presented a similar population of polymorphonuclear neutrophils (PMN), lymphocytes, and plasma cells. In both groups, it was observed that a large amount of the implanted material contained inflammatory cells inside and was surrounded by connective tissue. Further, an increase in the number of macrophages and giant cells in both groups was observed compared to the previous experimental period. Moreover, the LT group presented a trend that increased in these cells compared to the BG. There were no observable differences in the level of degradation or fragmentation of the membranes.

Also, the absence of necrosis, fatty infiltrate, and fibrosis was repeated after 21 days. A small increase in the number of blood vessels in the experimental groups (1 to 3 shoots) was observed.

#### 3.2.3. Sixty-Three Days Post-Implantation

After 63 days, the tissue in the Sham group was intact without any signs of inflammation (not shown); further, there was minimal mononuclear inflammatory infiltrate.

**Figure 4:**
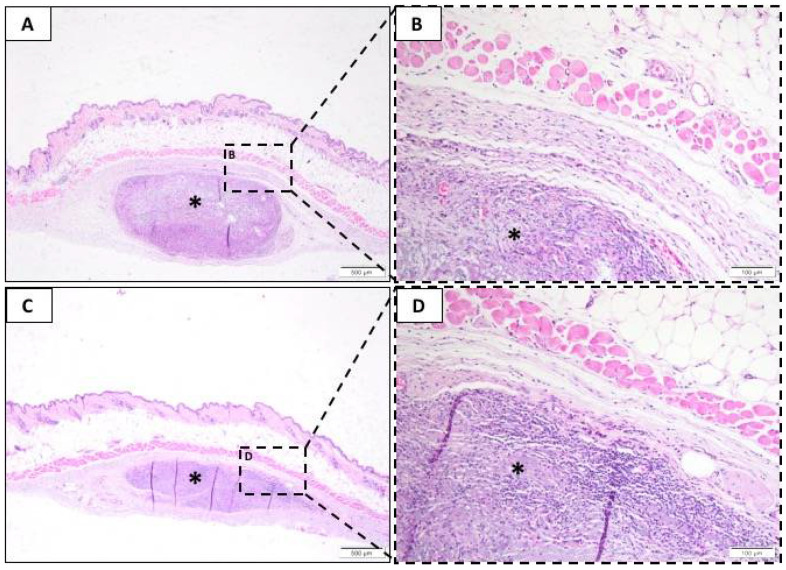
Representative photomicrographs of subcutaneous injury after 63 days. Histological section stained with hematoxylin/eosin from the incision region in the BG groups (**A**,**B**) and LT groups (**C**,**D**). The area occupied by the membrane is indicated by an asterisk (*). (**A**,**C**) 40× magnification, scale bar: 500 µm; (**B**,**D**) 200× magnification, scale bar: 100 µm.

Polymorphonuclear cells and plasma cells remained similar between BG (Figure 4A,B) and LT (Figure 4C,D). However, there was an increased number of lymphocytes and macrophages in BG compared to LT. Also, the number of macrophages surrounding the membrane increased compared to the previous (21-day) period at BG. Neovascularization presented as discrete for all groups, and necrosis, giant cells, fibrosis, and fatty infiltrate were absent.

For this period, we observed that the BG and LT groups presented different resorption patterns. There was a larger biodegradation area on the LT membrane than the BG membrane; both were permeated by an inflammatory infiltrate, predominantly of the mononuclear type.

### 3.3. Degree of Irritation Induced by Membranes According to ISO 10993-6:2016

**Figure 5:**
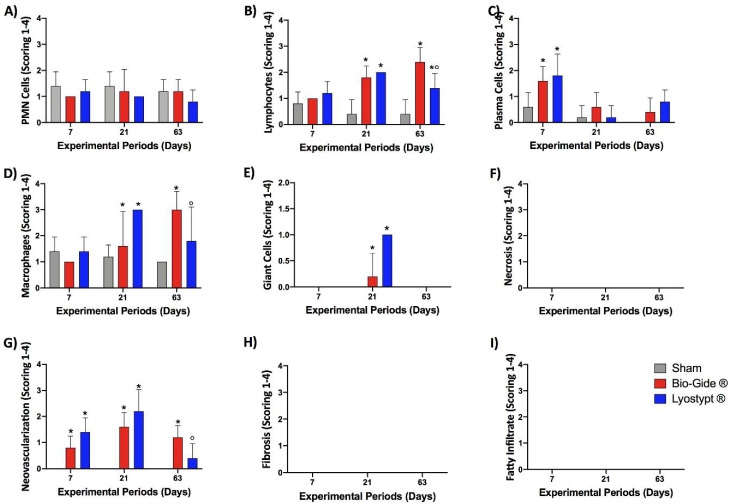
Inflammatory cells’ response (**A**–**F**) and overall tissue reaction (**G**–**I**) of Sham, Bio-Gide^®^, and Lyostypt^®^ groups after 7, 21, and 63 days of implantation. Data +/− standard deviation; *p* < 0.05; (*) represents a significant difference when compared to the Sham group; (°) represents significantly greater when compared to the Bio-Gide^®^ group.

**Figure 6:**
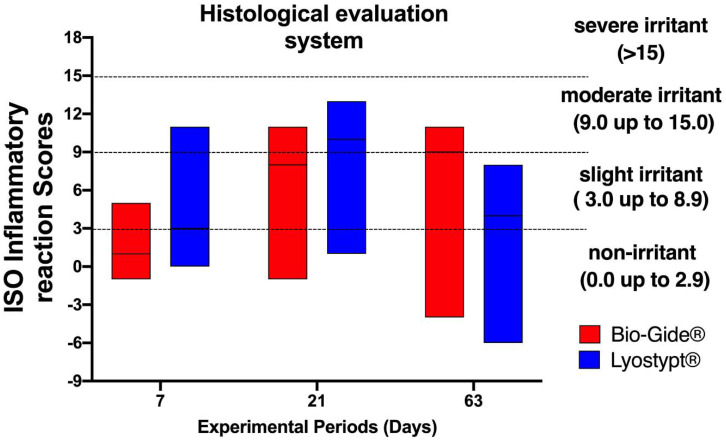
Score of inflammatory reaction according to ISO 10993-6:2016 of Bio-Gide^®^ and Lyostypt^®^ groups after 7, 21, and 63 days of implantation. The values obtained from Bio-Gide^®^ and Lyostypt^®^ groups were subtracted from the control (Sham group). The results are presented in the box as median, minimum, and maximum. The groups were ranked according to the criteria in ISO 10993-6:2016: non-irritant (0.0 to 2.9), slight irritant (3.0 to 8.9), moderate irritant (9.0 to 15.0), and severe irritant (>15).

The semiquantitative evaluation of implanted membranes was conducted according to ISO standard to compare the biological response of inflammatory cells (Figure 5A–F) and overall tissue reaction (Figure 5G–I) with the final calculated outcomes reported in Figure 6. The obtained values from LT and the BG membranes were subtracted by sham results. From the median of these findings the scores for each experimental group and period were classified. The Supplementary Tables S1–S3 present the total values.

The BG group, at the first experimental period, showed a median value of 1, classified as non-irritant. After 21 days, this group presented a peak of inflammatory cells like lymphocytes, macrophages, and giant cells, and the median value increased to 8, a slight irritant. At day 63, a moderate irritant (median = 9) was characterized mainly by an increased number of macrophage cells.

The LT group presented a slight irritant reaction (median = 3) seven days after surgery. At 21 days, an increase in the number of lymphocytes, macrophages, and giant cells classified this group as moderate irritant (median = 10). This pattern did not persist in the last experimental period, and the group was classified as slight irritant (median = 4).

## 4. Discussion

This study focused on the properties of two commercially available collagen membranes post-implantation in mice subcutaneous tissue. The biocompatibility, biodegradability, cellular reactions, and chronology of events were investigated in vivo. We hypothesized that the inflammatory cellular responses elicited by the LT membrane would be similar to those obtained by the BG membrane and, further, that both of these membranes would elicit the same reaction to the Sham group (a normal physiological inflammatory response). Additionally, the LT membrane, despite being a local hemostatic, could also be a barrier for guided tissue regeneration.

This study’s main limitation is that the studied membranes are indicated for guided bone regeneration to act as a barrier to avoid the population of non-osteogenic cells from the surrounding soft tissues in the bone defect. Thus, using these membranes in subcutaneous tissue would not evaluate the membrane’s effectiveness as a barrier. However, as shown in other studies [20,21], this model enables the assessment of the membranes’ safety (biocompatibility) and degradation (time the membrane remains in place). Thus, according to the ISO Standard [22], this study gave details on the recruitment of inflammatory cells, the formation of fibrous tissue and fatty infiltrate, and the degradation of membranes. Understanding how the tissue responds to the presence of membranes facilitates the knowledge of the biodegradation process.

The subcutaneous animal model was chosen in this study because of its ease in manipulation, the possibility for standardization, and the low cost of experimentation. Also, this model is recommended for evaluating medical and dental devices by the ISO Standard. The authors did not intend to extrapolate the results of this study for human use but understand the behavior in terms of biocompatibility and biodegradation of the membranes after implantation.

Differences were present in the morphology between the two membranes. The BG membrane is a non-cross-linked porcine membrane with type I and III collagen fibers. It does not contain any organic components or chemicals, and it has a bilayer structure composed of one compact side and one porous side, sterilized by gamma radiation [23]. The compact membrane layer has a smooth, condensed surface to protect it against unwanted cellular connections and tissue infiltration, while the porous layer allows cell invasion.

On the other hand, the LT membrane is non-cross-linked bovine type I collagen, obtained by the lyophilization process. It is widely used as a hemostatic agent and not as a barrier. It is also sterilized using gamma radiation and is clinically indicated for use in bleeding and situations that cause the accidental rupture of the schneiderian membrane.

The initial inflammatory response, which precedes the proliferative phenomena of scar repair, is acute. It is characterized by the mass migration of neutrophil polymorphonuclear leukocytes. However, this reaction is fleeting and lasts, on average, three days when there is a gradual replacement of neutrophils by mononuclear cells, mainly lymphocytes and plasma cells. Our main results showed that, in the seven days, the cellular reaction was similar. The LT group presented a slight increase in inflammatory cells, characterizing the response as slightly irritant and assuming that the responses were compatible with the physiological inflammatory process’ progress. Moreover, it was observed that the presence of lymphocytes and macrophages in the Sham group indicates a slight inflammatory reaction, similar to other groups. These findings can be interpreted using knowledge of the dynamics of the immuno-inflammatory phenomena in damaged tissue during the cicatricial repair [24].

However, it should be noted that the cellular scores for the animals with collagen membranes did not seem to be associated with the physicochemical composition of the membrane—they were related to the mechanical protection of the wound. This association reduces microbial contamination and facilitates the commencement and completeness of cicatricial repair [25]. Collagen membranes are reabsorbed in vivo by the enzymatic activity of infiltration by macrophages and polymorphonuclear leukocytes [26].

For the 21-day period, the appearance of giant cells was observed with a relatively high number of macrophages, demonstrating the early process of membrane degradation, mainly in the LT group. Other studies have also indicated that membranes’ morphological, physical, and chemical characteristics are considered important in modulating cellular events. These include protein adsorption and adhesion, fusion of macrophages to form giant foreign-body cells, and release of cytokines produced by these cells [27,28]. However, after the implantation of collagen membranes, which induce an inflammatory response, there may also be regulatory mechanisms to inhibit or even selectively limit this response and repair damaged tissue.

The 63 days was characterized by the maintenance of mononuclear infiltrate in both test groups, suggesting continuity in the process of membrane degradation. Further, the LT membrane demonstrated a larger biodegradation area, which was confirmed using light-field microscopy. Recent experimental research has shown that collagen membranes’ degradation can commence 4 to 28 days after installation [12,29]. This has been confirmed in several other studies; however, some chemically modified collagen membranes exhibit prolonged integrity with no severe inflammatory response [30]. In guided bone regeneration, a minimum period of 3 to 4 weeks is required to ensure the cells’ repopulation and maturation that form the bone matrix. Therefore, a collagen membrane must maintain integrity for this period at a minimum. This makes it clear that the duration of membrane integrity is likely to be a key factor for the formation and maturation of new bone in membrane-protected defects [30].

Processing techniques influence the properties of collagen significantly. Different types of cross-linking can lead to changes in the reabsorption rate of membranes, affecting their biological durability. Exposure to ultraviolet radiation, ethylene dioxide, glutaraldehyde, hexamethylene diisocyanate, or diphenyl-phosphorylazide retards the biodegradation process, while using different types of cross-linking does not change this pattern remarkably. However, in many cases, there is an increase in the inflammatory response and a reduction in tissue integration [31].

However, other studies demonstrate that cross-linked and non-cross-linked membranes are resistant to tissue degradation and remain stable for long periods. However, none of these membranes were resistant to degradation when exposed to the buccal medium [26]. Collagen membranes do not appear to be associated with a significant local inflammatory response or a systemic immune response. Thus, they seem to be well tolerated by the body, making them useful in various regeneration types [26].

Differences between the findings of this study and similar ones in literature justify divergences in the scores of inflammatory results. It is known that degradation and inflammatory-response characteristics differ between in vitro and in vivo studies, experimental sites [32,33], the chosen animal model, and the origin of the membrane used [34]. As used in this study, models with mice are commonly used for biocompatibility and biodegradation assays to investigate biomaterials. They provide a highly vascularized tissue area with easy access to the tissue layer. Further, connective tissue is out of reach of the animals’ paws [35].

More studies are required to evaluate the biocompatibility and biodegradation in different models that mimic the human oral cavity, to obtain more reliable and predictable data [36,37].

## 5. Conclusions

With the results obtained, it was possible to conclude that the biodegradation of the Lyostypt^®^ membrane occurred in a larger area compared to the Bio-Gide^®^ membrane after 63 days. Both membranes presented as biocompatible in subcutaneous mice. After guided bone regeneration procedures, bone regeneration follows a specific sequence of events that occur 2–3 months post-surgery. According to our results, the Bio-Gide^®^ group is well indicated for GBR as it lasts for at least nine weeks after implantation.

## Supplementary Materials

The following are available online at https://www.mdpi.com/2077-0375/10/12/423/s1, Table S1: Semiquantitative evaluation seven days post-implantation. The collected data from the semiquantitative analysis were used to evaluate the tissue reaction and biocompatibility, as suggested in ISO 10993-6. The values for each animal are a median of 10 sections evaluated per animal. Table S2: Semiquantitative evaluation 21 days post-implantation. The collected data from the semiquantitative analysis were used to evaluate the tissue reaction and biocompatibility, as suggested in ISO 10993-6. The values for each animal are a median of 10 sections evaluated per animal. Table S3: Semiquantitative evaluation 63 days post-implantation. The collected data from the semiquantitative analysis were used to evaluate the tissue reaction and biocompatibility, as suggested in ISO 10993-6. The values for each animal are a median of 10 sections evaluated per animal.
