# Supplementary material for: In Vivo Comparative Evaluation of Biocompatibility and Biodegradation of Bovine and Porcine Collagen Membranes
Source: Membranes (Basel). 2020 Dec 15;10(12):423. doi: 10.3390/membranes10120423 (PMC7765348; doi:10.3390/membranes10120423)
Supplement: Supplementary file 1 [file membranes-10-00423-s001.pdf]

membranes

In Vivo Comparative Evaluation of Biocompatibility and Biodegradation of Bovine and Porcine Collagen Membranes

Abdu Mansur Dacache Neto [1], Suelen Cristina Sartoretto [2,3,4], Isabelle Martins Duarte [5], Rodrigo Figueiredo de Brito Resende [3,6], Adriana Terezinha Neves Novellino Alves [7], Carlos Fernando de Almeida Barros Mourão [8], Jose Calasans-Maia [9], Pietro Montemezzi [10], Gilson Coutinho Tristão [11] and Mônica Diuana Calasans-Maia [4,6,*]

1 Graduate Program, Dentistry School, Universidade Federal Fluminense, Niteroi 24020-140, RJ, Brazil; abdudacache@globo.com (A.M.D.N.)
2 Oral Surgery Department, Dentistry School, Universidade Veiga de Almeida, Rio de Janeiro 20271-020, RJ, Brazil; susartoretto@hotmail.com (S.C.S.)
3 Oral Surgery Department, Dentistry School, Universidade Iguaçu, Nova Iguaçu 26260-045, RJ, Brazil; resende.r@hotmail.com (R.F.D.B.R.);
4 Clinical Research Laboratory in Dentistry, Universidade Federal Fluminense, Niteroi 24020-140, RJ, Brazil; monicacalasansmaia@gmail.com (M.D.C.-M.)
5 Post-Graduation Program in Dentistry, Universidade Veiga de Almeida, Rio de Janeiro 20271-020, RJ, Brazil; marcliodonto@terra.com.br (I.M.D.)
6 Oral Surgery Department, Universidade Federal Fluminense, Niteroi 24020-140, RJ, Brazil;
7 Oral Diagnosis Department, Universidade Federal Fluminense, Niteroi 24020-140, RJ, Brazil; aterezinhanovellino@gmail.com (A.T.N.N.A.)
8 Post-Graduation Program in Biotechnology, Universidade Federal Fluminense, Niteroi 24020-140, RJ, Brazil; mouraocf@gmail.com (C.F.D.A.B.M.)
9 Orthodontics Department, Universidade Federal Fluminense, Niteroi 24020-140, RJ, Brazil; josecalasans@id.uff.br (J.C.-M.)
10 Private Practice, 24128 Bergamo, Italy; m.montemezzi@libero.it (P.M.)
11 Periodontics Department, Universidade Federal Fluminense, Niteroi 24020-140, RJ, Brazil; gilsontb@vm.uff.br (G.C.T.)
* Correspondence: monicacalasansmaia@gmail.com; Tel.:+55-21-98153-5884

Table S1. Semiquantitative evaluation seven days post-implantation. The collected data from the semiquantitative analysis were used to evaluate the tissue reaction and biocompatibility, as suggested in ISO 10993-6. The values for each animal are a median of 10 sections evaluated per animal.

**7 days**	**SHAM**					**BioGide®**					**Lyostypt®**				
**Animals**	**1**	**2**	**3**	**4**	**5**	**1**	**2**	**3**	**4**	**5**	**1**	**2**	**3**	**4**	**5**
**Polymorphonuclear**	**1**	**2**	**1**	1	2	1	1	1	1	1	1	1	1	1	2
Lymphocytes	1	1	1	0	1	1	1	1	1	1	1	1	1	1	2
Plasma Cells	1	0	0	1	1	2	1	2	2	1	2	1	2	1	3
Macrophages	2	1	1	2	1	1	1	1	1	1	3	3	3	3	3
Giant cells	0	0	0	0	0	0	0	0	0	0	1	2	1	1	2
Necrosis	0	0	0	0	0	0	0	0	0	0	0	0	0	0	0
Score from inflammatory cells SUBTOTAL (X 2)	10	8	6	8	10	10	8	10	10	8	10	10	10	8	18
Neovascularization	0	0	0	0	0	1	1	1	1	0	1	1	2	1	2
Fibrosis	0	0	0	0	0	0	0	0	0	0	0	0	0	0	0
Fatty infiltrate	0	0	0	0	0	0	0	0	0	0	0	0	0	0	0
Score from tissue response SUB-TOTAL	0	0	0	0	0	1	1	1	1	0	1	1	2	1	2
TOTAL	10	8	6	8	10	11	9	11	11	8	11	11	12	9	20
Avarege	8,4					10					12.6				
Result (Test–control)	---					1.6					4.2				

Table S2. Semiquantitative evaluation 21 days post-implantation. The collected data from the semiquantitative analysis were used to evaluate the tissue reaction and biocompatibility, as suggested in ISO 10993-6. The values for each animal are a median of 10 sections evaluated per animal.

7 days	SHAM					BioGide®					Lyostypt®				
Animals	1	2	3	4	5	1	2	3	4	5	1	2	3	4	5
Polymorphonuclear	2	1	1	1	2	0	1	1	2	2	1	1	1	1	1
Lymphocytes	0	0	1	1	0	1	2	2	2	2	2	2	2	2	2
Plasma Cells	0	0	0	0	1	1	1	1	0	0	0	0	1	0	0
Macrophages	1	1	1	1	2	0	1	1	3	3	3	3	3	3	3
Giant cells	0	0	0	0	0	0	1	0	0	0	1	1	1	1	1
Necrosis	0	0	0	0	0	0	0	0	0	0	0	0	0	0	0
Score from inflammatory cells SUBTOTAL (X 2)	6	4	6	6	10	4	12	10	14	14	14	14	16	14	14
Neovascularization	0	0	0	0	0	1	1	2	2	2	2	3	2	3	1
Fibrosis	0	0	0	0	0	0	0	0	0	0	0	0	0	0	0
Fatty infiltrate	0	0	0	0	0	0	0	0	0	0	0	0	0	0	0
Score from tissue response SUB-TOTAL	0	0	0	0	0	1	1	2	2	2	2	3	2	3	1
TOTAL	6	4	6	6	10	5	13	12	16	16	16	17	18	17	15
Avarege	6,4					12.4					16.6				
Result (Test – control)	---					6					10.2				

Table S3. Semiquantitative evaluation 63 days post-implantation. The collected data from the semiquantitative analysis were used to evaluate the tissue reaction and biocompatibility, as suggested in ISO 10993-6. The values for each animal are a median of 10 sections evaluated per animal.

7 days	SHAM					BioGide®					Lyostypt®				
Animals	1	2	3	4	5	1	2	3	4	5	1	2	3	4	5
Polymorphonuclear	1	2	1	1	1	1	1	1	2	1	0	1	1	1	1
Lymphocytes	1	0	0	0	1	2	2	2	2	2	1	2	2	1	1
Plasma Cells	0	0	0	0	0	1	0	0	0	1	1	0	1	1	1
Macrophages	1	1	1	1	1	3	3	3	2	3	0	2	1	3	3
Giant cells	0	0	0	0	0	0	0	0	0	0	0	0	0	0	0
Necrosis	0	0	0	0	0	0	0	0	0	0	0	0	0	0	0
Score from inflammatory cells SUBTOTAL (X 2)	6	6	4	4	6	14	12	12	12	14	4	10	10	12	12
Neovascularization	0	0	0	0	0	1	1	1	1	2	1	0	1	0	0
Fibrosis	0	0	0	0	0	0	0	0	0	0	0	0	0	0	0
Fatty infiltrate	0	0	0	0	0	0	0	0	0	0	0	0	0	0	0
Score from tissue response SUB-TOTAL	0	0	0	0	0	1	1	1	1	2	1	0	1	0	0
TOTAL	6	6	4	4	6	15	13	13	13	16	5	10	11	12	12
Avarege				5.2				15.2					10		
Result (Test–control)				---				10					4.8		
